# Assessment of changes in gaze patterns during training in point-of-care ultrasound

**DOI:** 10.1186/s12909-022-03680-5

**Published:** 2022-09-02

**Authors:** Alice H. Y. Chan, Wei Feng Lee, Pascal W. M. Van Gerven, Jordan Chenkin

**Affiliations:** 1 Department of Emergency Medicine, Sunnybrook Health Sciences Center, University of Toronto, 2075 Bayview Avenue, AG245, Toronto, ON M4N 3M5 Canada; 2 Department of Emergency Medicine, Ng Teng Fong General Hospital, 1 Jurong East Street 21, Singapore, Singapore 609606; 3 Department of Educational Development and Research, School of Health Professions Education, Faculty of Health, Medicine and Life Sciences, Maastricht University, Maastricht, Netherlands

**Keywords:** Point-of-care ultrasound, Gaze patterns, Competency assessment

## Abstract

**Background:**

Point-of-care ultrasound (POCUS) is a core skill in emergency medicine (EM), however, there is a lack of objective competency measures. Eye-tracking technology is a potentially useful assessment tool, as gaze patterns can reliably discriminate between experts and novices across medical specialties. We aim to determine if gaze metrics change in an independent and predictable manner during ultrasound training.

**Methods:**

A convenience sample of first-year residents from a single academic emergency department was recruited. Participants interpreted 16 ultrasound videos of the focused assessment with sonography for trauma (FAST) scan while their gaze patterns were recorded using a commercially available eye-tracking device. The intervention group then completed an introductory ultrasound course whereas the control group received no additional education. The gaze assessment was subsequently repeated. The primary outcome was total gaze duration on the area of interest (AOI). Secondary outcomes included time to fixation, mean duration of first fixation and mean number of fixations on the AOI.

**Results:**

10 EM residents in the intervention group and 10 non-EM residents in the control group completed the study. After training, there was an 8.8 s increase in the total gaze time on the AOI in the intervention group compared to a 4.0 s decrease in the control group (*p* = .03). EM residents were also 3.8 s quicker to fixate on the AOI whereas the control group became 2.5 s slower (*p* = .04). There were no significant interactions on the number of fixations (0.43 vs. 0.18, *p* = .65) or duration of first fixation on the AOI (0.02 s vs. 0.06 s, *p* = .63).

**Conclusions:**

There are significant and quantifiable changes in gaze metrics, which occur with incremental learning after an ultrasound course. Further research is needed to validate the serial use of eye-tracking technology in following a learner’s progress toward competency in point-of-care ultrasound image interpretation.

## Background

The use of point-of-care ultrasound (POCUS) is widespread in emergency departments [1]. This adjunct to the physical exam helps the physician ‘see inside’ the patient at the bedside to answer focused clinical questions and guide appropriate management [2]. There are many advantages of POCUS over other common imaging modalities such as X-ray and computed tomography (CT). POCUS is portable, repeatable, cost-effective and carries no risk of ionizing radiation. From timely diagnoses, to safer procedures, to reduced length of stay, POCUS significantly improves patient outcomes and overall quality of care [3, 4]. Learning emergency ultrasound, therefore, has become a standard part of the core curriculum of most emergency medicine residency training programs worldwide [4–7].

Objective measures are needed to guide the novice’s pathway to expertise. However, current assessment methods for POCUS competency are subjective, costly and labour-intensive. These traditionally include a combination of direct observation of scans, followed by written, visual and practical examinations. The significant assessment burden on faculty is a challenge for most programs, leading to a push for new automated tools to measure competency.

Eye-tracking is a promising technology in this area of ultrasound education. It involves the continuous measurement of an individual’s eye position and movement over time [8]. The path of gaze with respect to the location, order and duration of fixation, reflects one’s attention allocation. While these metrics do not correlate directly with cognition, they can provide valuable insight into a learner’s thought processes [9, 10].

Prior studies in the fields of anesthesiology, general surgery and radiology have provided preliminary evidence for gaze analysis to differentiate between POCUS skill levels [11–13]. Experts consistently demonstrated more efficient gaze paths compared to novices. They spend less time identifying sonoanatomy and were quicker to fixate on pathologic findings due to superior pattern recognition skills [13, 14]. They also fixated longer on structures of interest and devoted less attention to irrelevant visual stimuli [14]. These differences in gaze patterns based on expertise level were also noted for ultrasound-guided procedures, including venipunctures and nerve blocks [14, 15]. Richstone et al. established the predictive utility of gaze patterns, which alone can reliability discern expert from non-expert surgeons with 92% accuracy [16]. Similarly, in our recent proof of concept study, we showed that experts have a faster fixation and longer gaze time on the area of interest (AOI) relative to novices during interpretation of focused assessment using sonography in trauma (FAST) scans [17].

While eye tracking has been shown to discriminate between the extremes of ultrasound competency, there is limited literature on the short-term evolution of gaze metrics with learning. As with other procedural skills in medicine, POCUS mastery is based on time, experience and iterative practice. To date, it is unknown whether there is a delay or lag time in the change to gaze parameters with incremental learning. We designed this study to determine whether gaze metrics change dynamically when interpretating ultrasound clips before and after an introductory POCUS course. If gaze metrics are proven to correlate with learning, this work will provide further construct validity for the use of eye tracking in the assessment for POCUS competency.

## Methods

### Study design

We performed a quasi-experimental study in an urban university-affiliated tertiary care hospital in Toronto, Ontario with more than 65 000 annual ED visits. There is an established emergency medicine residency program and emergency ultrasound fellowship program. Approval for this study was granted by the Sunnybrook Health Sciences Center research ethics board. Written informed consent was obtained from all enrolled subjects.

### Study setting and population

A convenience sample of junior residents was enrolled from July 2020 to January 2021. Inclusion criteria were first year emergency medicine and off-service (i.e. non-emergency medicine) residents with no prior formal ultrasound training. Exclusion criteria were refusal to participate and failure of the calibration process with the eye-tracking device. A survey on basic demographics and prior experience with the FAST ultrasound application was collected. All data and results were anonymized and kept confidential.

### Study protocol

**Fig. 1:**
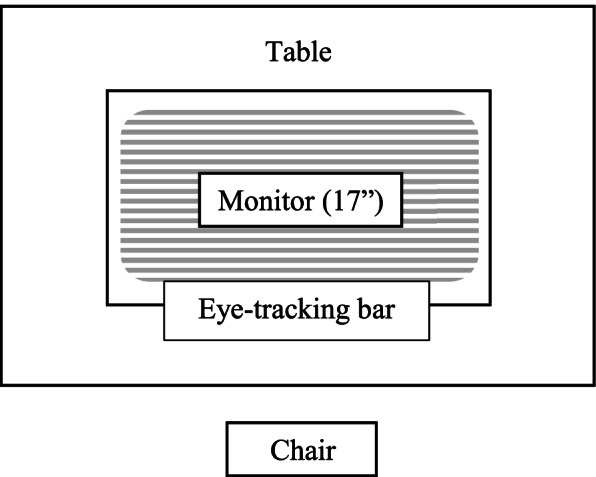
Schematic of room setup from an aerial point of view

For gaze tracking, we used the Tobii Nano Pro (Tobii, Karlsrovagen, Sweden), a commercially available screen-based eye tracker. This device has a sampling frequency of 60 Hz, precision of 0.1° and accuracy of 0.3° at optimal conditions. Each participant was seated in front of a 17″ monitor with the eye tracker mounted at the bottom of the screen (Fig. 1). The monitor was set at a constant brightness and contrast setting while the overhead lights stayed on in the room during data collection.

Calibration was performed using the eye-tracking software (Tobii Pro Lab, Version 1.130) by having the participant focus on a traveling white target across the screen. If calibration failed, adjustments were made to the positioning of the device and the individual. If re-calibration remained unsuccessful after three attempts, the participant were removed from the study.

Emergency medicine residents were assigned to the intervention arm, while the non-emergency medicine residents were assigned to the control arm. All participants completed a baseline assessment, in which they viewed 16 ultrasound clips that were part of the FAST application, including both normal and pathologic findings. These clips were of variable difficulty and included views of the right upper quadrant, left upper quadrant, pelvis and subxiphoid cardiac scans. Areas of interest were jointly determined by consensus by two faculty members who are certified ultrasound instructors and POCUS experts. Each video was four seconds long and looped twice. Participants were not able to pause or stop the videos. Eye motions and gaze fixations were recorded for analysis. Following each video, participants were asked to interpret the scan as either positive, negative or indeterminate for intraperitoneal free fluid or pericardial effusion. The correct responses were not disclosed to prevent introducing bias on repeat assessment.

Following the baseline testing, the intervention group attended an 8-h introductory POCUS course with didactic teaching and hands-on practice. Each resident completed an average of 20 supervised scans per core POCUS application (subxiphoid cardiac, aortic, abdominal, first trimester obstetrics, pleural effusion and pneumothorax) within this time. The control group did not receive any further POCUS education during this period.

Both groups then performed a repeat assessment of the 16 ultrasound clips, with the order of the clips modified to reduce serial position effect. Participants also completed a written examination (consisting of 20 POCUS-related multiple choice questions) to evaluate knowledge base at the beginning and the end of the study. Content included basic knobology, artifacts, pitfalls, image generation, image interpretation and image optimization. These questions differed from baseline to post-training but were intentionally set at a similar level of difficulty.

### Outcome measures

The primary outcome was total gaze time on the AOI (seconds). This was based on our pilot study [17] and other studies using eye-tracking in ultrasound-guided regional anesthesia [13, 14], which demonstrated significantly longer fixation on the AOI among experts compared to novices. Secondary outcomes included time to first fixation on the AOI (seconds), mean duration of first fixation on the AOI (seconds) and mean number of fixations on the AOI. These gaze metrics were generated using the Tobii Pro Lab software. We also assessed changes in scores on the written examination and visual examination.

### Data analysis

Sample size was calculated based on the effect size from our pilot study comparing experts and novices. A total of 20 participants (10 per group) were required to establish a 35% difference before and after POCUS training with a power of 80%. Gaze parameters were automatically recorded by the eye-tracking software. Data were imported into SPSS (Version 25) for statistical analysis. Repeated-measures analysis of variance (ANOVA) was used to account for baseline imbalances between the groups. Results were analyzed for the main effects of within-group (pre- and post-course), between-group (intervention and control) and their interaction (within-group by between-group). *p*-values of less than 0.05 were considered statistically significant.

The reliability of each gaze parameter was assessed using Cronbach’s alpha. This is a measure of internal consistency that describes the extent to which all the items in a test measure the same construct. A value above 0.80 is good, a value between 0.71–0.80 is reasonable, a value between 0.51–0.70 is questionable or poor, and a value below 0.50 is unacceptable [18]. The interrelatedness of each gaze parameter within the test was analyzed separately to support its validity.

## Results

A convenience sample of 20 participants (10 in the intervention arm and 10 in the control arm) were recruited in this study. There were no exclusions. All participants completed the study procedures. Characteristics of the study sample are shown in Table 1 with interquartile range (IQR) where applicable.

**Table 1:** Baseline characteristics of participants

	Intervention (*n* = 10)	Control (*n* = 10)
Sex		
Male	8	4
Female	2	6
Median age in years (IQR)	27.5 (25.8–30.0)	26.0 (25.8–30.0)
Median number of supervised FAST scans (IQR)	5.0 (3.0–12.5)	0.0 (0.0–1.0)

Repeated-measures ANOVA demonstrated interactions for the primary outcome (total gaze duration on the AOI) and the secondary outcome (time to fixation on the AOI) as a result of the POCUS course. The intervention group had an 8.8 s increase in the total gaze time on the AOI compared to a 4.0 s decrease in the control group (*p* = 0.03). The intervention group was also 3.8 s quicker to fixate on the AOI whereas the control group became 2.5 s slower (*p* = 0.04).

**Fig. 2:**
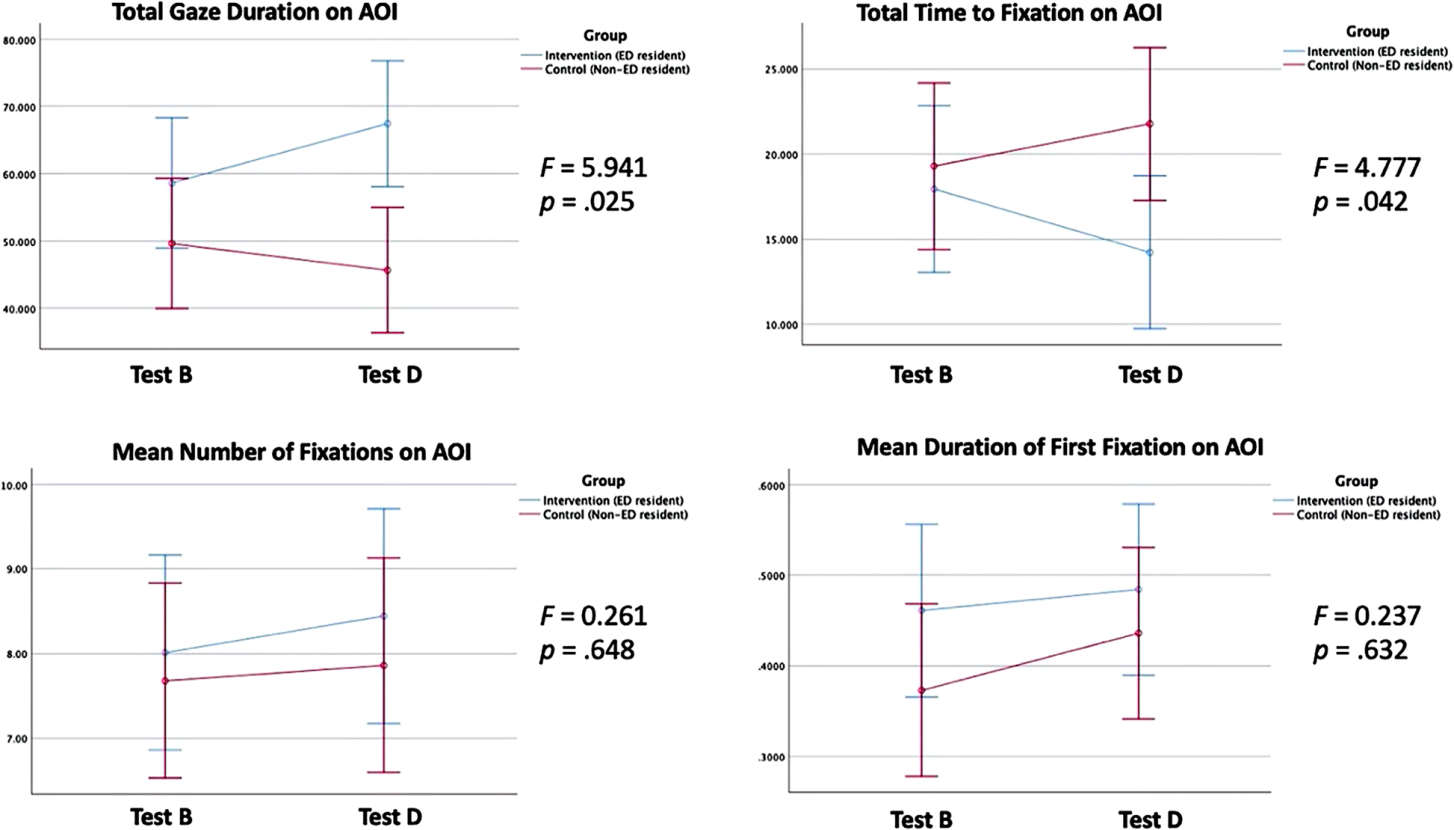
Plots of eye-tracking parameters

There were no significant interactions on the numbers of fixations on the AOI (0.43 vs. 0.18, *p* = 0.65) or the duration of first fixation on the AOI (0.02 vs. 0.06, *p* = 0.63) after the POCUS course. The results are summarized in Table 2 and Fig. 2.

**Table 2:** Results of eye-tracking parameters

Eye tracking metric	Intervention			Control			*p*-value
	Pre-course	Post-course	Mean difference	Pre	Post	Mean difference	
Total gaze duration on AOI (seconds)	58.6	67.4	+ 8.80 (15.0%)	49.6	45.6	- 4.00 (-8.1%)	**.03**
Total time to fixation on AOI (seconds)	18.0	14.2	- 3.80 (-21.1%)	19.3	21.8	+ 2.50 (13.0%)	**.04**
Mean number of fixations on AOI	8.01	8.44	+ 0.43 (5.4%)	7.68	7.86	+ 0.18 (2.3%)	.65
Mean duration of first fixation on AOI (seconds)	0.461	0.484	+ 0.02 (5.0%)	0.373	0.436	+ 0.06 (16.9%)	.63

*AOI* Area of interest

Total gaze time on the AOI and mean number of fixations on the AOI showed good internal consistency, with $\alpha$ = 0.81 and $\alpha$ = 0.88 respectively. Total time to fixation on the AOI demonstrated moderate internal consistency with $\alpha$ = 0.72. Mean duration of first fixation on the AOI demonstrated low internal consistency with $\alpha$ < 0.50.

For both timepoints in the study, the intervention group scored higher on the examinations compared to the control group. However, the ultrasound course was not significantly associated with performance improvement between the groups on the written examination (5.5% vs. 0%, *p* = 0.56) nor the visual examination (12.2% vs. 2.7%, *p* = 0.19). The results are summarized in Table 3.

**Table 3:** Results of written and visual examinations

Eye tracking metric	Intervention			Control			*p*-value
	Pre-course	Post-course	Mean difference	Pre	Post	Mean difference	
Written exam (%)	73.0	77.0	+ 4.00 (5.5%)	58.5	58.5	0.00 (0%)	.56
Visual exam (%)	71.3	80.0	+ 8.70 (12.2%)	70.0	71.9	+ 1.9 (2.7%)	.19

## Discussion

In this study, we found that there are meaningful and quantifiable changes in gaze metrics that occur after a single introductory ultrasound course. The intervention group fixated significantly quicker and longer on the area of interest compared to the control group. The total gaze time and the time to first fixation on the AOI demonstrated good internal reliability as per Cronbach’s alpha. These findings can be explained by several factors. As residents gain expertise, they may be quicker to recognize sonoanatomy of interest and to spend a longer time in the highest yield location. In addition, they have a more focused gaze pattern with less distracted time spent outside of the AOI. The evolution in eye-tracking metrics with learning has potential to provide objective competency measures during the training period.

Our findings are consistent with the results of prior eye-tracking work, including our pilot study, in which the experts exhibited more efficient and effective gaze behavior compared to the novices. A recent study by Bell et al. [19] explored the differences in gaze patterns between emergency medicine residents and emergency ultrasound fellows when viewing a standardized FAST video. Gaze fixations over nine critical anatomic regions of interest (ROIs) were identified. 100% of fellows visually interrogated every ROI whereas only 58% of residents viewed all nine ROIs. Five ROIs were identified over which at least one resident sonographer did not have a gaze fixation. Harrison et al. [14] showed that experts had longer fixations on the AOI when performing procedures, specifically ultrasound-guided paravertebral nerve blocks. Wilson et al. [20] showed that novices can be trained to gaze like an expert by studying an expert’s gaze behavior. This type of training resulted in faster completion of a simulated laparoscopic surgical technique compared to more traditional methods of training.

At present, there is no consensus on how best to assess competency in POCUS, and in turn, when to grant a learner the ability to practice POCUS independently. Most methods are subjective, time-intensive and not aligned with the recent shift to competency-based education models [21]. There are only a few prospective studies using objective tools to assess for expertise in this area. They focused exclusively on the image generation component of the FAST examination using hand motion analysis (HMA), which measures the motion of a participant’s hand while completing a task. In a surgical study, Ziesmann et al. [22] applied HMA technology to twelve expert and twelve novice sonographers as they performed a FAST exam on a human model. Experts were observed to complete the FAST exam with fewer total movements and a shorter path length of travel compared to novices. Bell et al. [23] studied whether there are measurable differences in hand motions with acquisition of expertise. They recruited 15 emergency medicine residents with limited POCUS experience. Probe motion was analyzed before and after the completion of at least 50 supervised FAST scans. They found that with experience, the scan was completed in less time with less motion and more imaged AOIs. Chin et al. further established construct validity for HMA as a proficiency assessment tool in ultrasound-guided peripheral nerve blockade. They demonstrated excellent correlation for junior residents and experienced anesthesiologists between HMA parameters, a task-specific checklist and a global rating score [24]. This is akin to our study objective to demonstrate eye-tracking technology as an objective competency measure for the image interpretation domain of the FAST exam. The use of eye-tracking and HMA together to follow image generation and image interpretation, which happens simultaneously in practice to produce clinically relevant information, has not been studied to date.

Using eye-tracking for assessment has many advantages. The eye-tracking device is relatively inexpensive and portable, as it can be attached to any laptop and set up in any location. Learners can obtain individualized feedback, quantitatively with gaze metrics or qualitatively with gaze heat maps and real-time video review. For educators, attentional behaviors can help identify knowledge gaps and allow early remediation. A learner who fixates correctly but misinterprets an AOI may need teaching around positive findings, whereas a learner who has an unfocused gaze pattern may need to study sonoanatomy. On a systems level, gaze behavior can be used to assess the impact of educational programs and improve curriculum design. Interestingly, in our study, we demonstrated a significant change in gaze metrics after the ultrasound course, but there was no parallel improvement in performance on the written and visual exam. This suggests that eye metrics may function as an early marker of learning in point-of-care ultrasound image interpretation.

## Limitations

This study had a number of important limitations. First, it was performed at a single academic tertiary care center with a small sample size. Second, the intervention and control groups were drawn from different resident populations. We were unable to randomize emergency and non-emergency medicine residents to the groups as the introductory POCUS course is only held once annually for first-year emergency medicine residents. Third, there may have been a selection bias leading to an over-representation of residents in the control arm with a relatively strong baseline interest in POCUS. Fourth, we selected FAST videos with a single dynamic AOI per clip. However, multiple AOIs often appear concurrently (i.e. free fluid in the caudal tip of the liver and the hepatorenal interface), which would make analysis more complex. Fifth, there may be a Hawthorne effect, in which participants modify their gaze behavior in response to their awareness of being observed. Finally, this study only assessed for the surrogate measures of image interpretation (i.e. gaze metrics relative to AOI) and cannot comment on the validity of gaze patterns for assessing image generation.

## Conclusions

Gaze pattern analysis is a promising tool for assessing learning in the image interpretation component of point-of-care ultrasound. We determined that a significant change in gaze metrics, specifically shorter time to first fixation on the AOI and longer total gaze time on the AOI, occurred for interpretation of focused assessment with sonography in trauma scans before and after completion of an ultrasound course. This study provides the groundwork for future research on the use of eye-tracking technology by creating objective benchmarks for POCUS performance as learners progress towards expertise.

## Data Availability

All data generated or analysed during this study are included in this published article.
